# Study on Autoimmune Thyroid Disorders in Chronic Spontaneous Urticaria at a Tertiary Care Central Rural Hospital in India

**DOI:** 10.7759/cureus.39398

**Published:** 2023-05-23

**Authors:** Meghana Pendam, Bhushan Madke, Adarshlata Singh, Sugat Jawade

**Affiliations:** 1 Dermatology, Venereology and Leprosy, Jawaharlal Nehru Medical College, Datta Meghe Institute of Higher Education & Research, Wardha, IND

**Keywords:** severity, clinical features, anti-tpo antibodies, autoimmune thyroid diseases, mast cells, chronic spontaneous urticaria

## Abstract

Background

Chronic spontaneous urticaria (CSU) is a medical condition characterized by the persistence of urticaria for more than six weeks, primarily caused by mast cell activation. Autoimmune thyroid diseases (AITDs) are the most common cause of dysfunction of the thyroid gland, and they are influenced by both genetic and environmental factors. Mast cell mediators have a significant role in the pathogenesis of CSU through two main pathways: the derangement of intracellular signaling pathways in mast cells and basophils and the production of autoantibodies against these cells. This study aimed to explore the association between AITDs and CSU by examining clinical features and measuring thyroid hormones and anti-thyroid peroxidase (anti-TPO) antibodies in patients.

Aim and objectives

The primary aims of this study are to investigate the prevalence and clinical characteristics of autoimmune thyroid disorders in patients with chronic spontaneous urticaria. The specific objectives are to evaluate the triiodothyronine (T3), tetraiodothyronine (T4), thyroid-stimulating hormone (TSH), and anti-thyroid peroxidase (anti-TPO) antibody levels in patients and controls and to explore the correlations between these parameters and the development and severity of chronic spontaneous urticaria.

Material and method

The present study was an observational investigation that enrolled 40 patients, consisting of 20 cases and 20 controls. The inclusion criteria involved patients of both sexes, aged above 18 years, who had chronic spontaneous urticaria and agreed to participate in the study with informed consent. Patients with other skin conditions lacking abnormal thyroid etiopathogenesis were also included. Exclusion criteria included patients with major systemic disease, uncontrolled medical or surgical illness, renal or hepatic disorders, and pregnant or lactating females. Patients with chronic spontaneous urticaria underwent a comprehensive clinical evaluation, and their urticaria severity was scored using an established scoring system. Blood samples were collected from both cases and controls for measuring T3, T4, TSH, and anti-TPO antibody levels. The anti-TPO antibody was processed using the enzyme-linked immunosorbent assay (ELISA) method. The autoimmune thyroid disease was screened by monitoring T3, T4, TSH, and anti-TPO antibody levels.

Results

There were significant variations observed in thyroid-stimulating hormone and anti-thyroperoxidase antibody levels. Among the cases analyzed, 40% demonstrated an urticaria severity score of one, while 25% reported a duration exceeding eight weeks. Additionally, 25% of patients experienced severe pruritus and intense wheals.

Conclusions

This research has discovered a robust association between serum anti-TPO antibodies and the occurrence of chronic spontaneous urticaria. To mitigate the potential for chronic spontaneous urticaria to lead to long-term morbidity, it is imperative to consider testing for serum anti-TPO antibodies in conjunction with primary thyroid markers, including T3, T4, and TSH.

## Introduction

Chronic spontaneous urticaria is a condition in which urticaria persists for more than six weeks [1]. The disease is primarily caused by mast cells, which release histamine upon activation, leading to vasodilation, plasma extravasation, and cell recruitment to the urticarial lesion. While various substances can stimulate mast cells in urticaria, their specific identification is often unclear [2]. The causes of chronic urticaria are diverse and can include autoimmune diseases, urticarial vasculitis, thyroid autoimmunity, connective tissue disorders, and infections [3,4]. Clinical presentations of chronic urticaria are varied, with wheals and associated systemic manifestations being the most common features [3,4].

Autoimmune thyroid disorders are a result of immune system imbalances. These disorders are mediated by organ-specific T cells and are characterized by lymphocyte infiltration in the thyroid parenchyma [5]. The clinical hallmarks of autoimmune thyroid diseases are thyrotoxicosis or hypothyroidism. Epidemiological data suggest genetic predisposition and environmental triggers are associated with disease onset [5]. Type one helper lymphocytes increase IFN-γ and CXCL10, which initiate the autoimmune process [6]. A significant correlation has been established between autoimmune thyroid disorders and chronic spontaneous urticaria, necessitating the evaluation of triiodothyronine (T3), tetraiodothyronine (T4), thyroid-stimulating hormone (TSH), and anti-thyroid peroxidase (anti-TPO) antibody levels in patients with chronic spontaneous urticaria.

## Materials and methods

Study setting and design

The case-control study was conducted at the Department of Dermatology, Venereology and Leprosy, Acharya Vinobha Bhave Rural Hospital, Jawaharlal Nehru Medical College, Sawangi (M), Wardha, over two years from October 2020 to December 2022.

Study population and sample size

20 clinically diagnosed cases of chronic spontaneous urticaria of both genders above 18 years and 20 controls attending the outpatient department of dermatology after considering various inclusion and exclusion criteria.

Sample characteristics (inclusion and exclusion criteria)

The study's inclusion criteria encompassed patients of both genders who were at least 18 years of age and willing to participate with informed consent. Specifically, the study focused on patients with chronic spontaneous urticaria but also included individuals with other skin conditions as long as they did not present any irregular thyroid gland function.

Conversely, exclusion criteria comprised patients with significant systemic diseases or uncontrolled medical or surgical conditions, individuals on anti-thyroid medications, patients with renal or hepatic disorders, and pregnant or lactating women.

Data collection process and method

Following the application of inclusion and exclusion criteria, patients diagnosed with chronic spontaneous urticaria and seeking treatment at the Department of Dermatology, Venereology, and Leprosy, AVBRH, Sawangi, Wardha were recruited for the study. Approval was obtained from the Institutional Ethical Committee (IEC), and all participants provided informed written consent in their native language voluntarily. Comprehensive clinical histories were collected, which included the patient's name, age, gender, family medical history, and past events. The patients with chronic spontaneous urticaria underwent a thorough clinical evaluation and were assessed based on a standardized urticaria severity scoring system [7].

T3, T4, TSH, and anti-TPO antibody levels were assessed via blood sampling using established protocols in both the cases and controls. The peripheral (cubital) vein was accessed to obtain the blood samples, which were then collected in red top vacutainers. The V5600 dry chemistry technique utilizing enhanced chemiluminescence was used to process the blood samples and measure the T3, T4, and TSH levels. Anti-TPO antibody levels were assessed using the enzyme-linked immunosorbent assay (ELISA) method. These measurements were used to screen for autoimmune thyroid disease [8].

Statistical analysis

The collected data were subjected to statistical analysis using IBM SPSS Statistics for Windows, Version 24.0 (Released 2016; IBM Corp., Armonk, New York, United States). Demographic information was gathered using a questionnaire designed by the researchers for the study. The mean value was computed and assigned a rating. Statistical significance was deemed attained if the p-value was less than 0.05 at a 95% confidence level.

Ethical consideration

Before participating in the study, every participant was provided with a detailed explanation regarding the concept and objectives of the research and subsequently provided their informed consent in writing. Confidentiality and privacy were explicitly assured to each participant. Moreover, the research protocol underwent rigorous review, was approved by the DMIMS (DU)/IEC/2020-21/9315, and was published in the Journal of Pharmaceutical Research International [9].

## Results

Table 1 comprises measurements taken from a sample of 20 subjects, divided equally into case and control groups, whose ages ranged between 18 and 60 years, with a mean age of 36.40 ± (11.03) for the case group and 32.95 ± (11.58) for the control group. The female gender comprised most of this study's subjects (55%), while males accounted for 45% of the case group. In contrast, the control group displayed an equal distribution of both genders, as shown in Table 2.

**Table 1:** Distribution of patients in two groups according to their age in years

Age Group(yrs)	Cases	Controls	ϗ2-value	P-Value
≤20 yrs	1(5%)	3(15%)	2.65	0.61
21-30 yrs	6(30%)	7(35%)		
31-40 yrs	5(25%)	6(30%)		
41-50 yrs	5(25%)	2(10%)		
51-60 yrs	3(15%)	2(10%)		
Total	20(100%)	20(100%)		
Mean±SD	36.40±11.03	32.95±11.58		
Range	20-55 yrs	18-60 yrs		

**Table 2:** Distribution of patients in two groups according to their gender

Gender	Cases	Controls	ϗ2-value	P-Value
Male	9(45%)	10(50%)	0.10	0.75
Female	11(55%)	10(50%)		
Total	20(100%)	20(100%)		

The statistical analysis of Table 3 and Table 4 indicates that the estimated values of triiodothyronine and tetraiodothyronine for the case and control groups did not reach statistical significance, as indicated by p-values of 0.62 and 0.41, respectively.

**Table 3:** Comparison of triiodothyronine (T3) values in two groups

Group	N	Mean	Std. Deviation	Std. Error Mean	t-value	P-Value
Cases	20	1.44	0.28	0.06	0.09	0.62
Controls	20	1.43	0.22	0.05		

**Table 4:** Comparison of tetraiodothyronine (T4) values in two groups

Group	N	Mean	Std. Deviation	Std. Error Mean	t-value	P-Value
Cases	20	10.77	2.66	0.59	0.80	0.41
Controls	20	10.12	2.10	0.46		

The findings from Tables 5 and 6 indicate a statistically significant difference between the case and control groups concerning thyroid stimulating hormone and anti-thyroperoxidase antibody levels (p= 0.034 and p= 0.039, respectively).

**Table 5:** Comparison of thyroid stimulating hormone (TSH) values in two groups

Group	N	Mean	Std. Deviation	Std. Error Mean	t-value	P-Value
Cases	20	2.91	2.52	0.56	2.19	0.034
Controls	20	1.62	0.77	0.17		

**Table 6:** Comparison of anti-thyroperoxidase antibody (anti-TPO antibody) values in both cases and control groups

Group	N	Mean	Std. Deviation	Std. Error Mean	t-value	P-Value
Cases	20	18.28	8.98	2	2.13	0.039
Controls	20	13.57	4	0.89		

According to the distribution of participants in the case group, as per their urticaria severity score, it was noted that 40% of the instances had a score of one, 35% had a score of two, and 25% had a score of three, as presented in Table 7.

**Table 7:** Distribution of patients according to urticaria severity score in the case group

Urticaria Severity Score	No of Patients	Percentage
Score 1	8	40
Score 2	7	35
Score 3	5	25
Total	20	100

Upon stratification of the cases based on their duration in weeks, it was observed that 35% of the cases lasted for six weeks, 40% lasted for seven weeks, and 25% persisted for over eight weeks. The mean value for the duration of the cases was determined to be 6.90 ± 0.78 (6-8 weeks), as presented in Table 8.

**Table 8:** Distribution of patients according to duration (weeks) in the case group

Duration (weeks)	No of Patients	Percentage
6 weeks	7	35
7 weeks	8	40
8 weeks	5	25
Total	20	100
Mean±SD	6.90±0.78(6-8 weeks)	

Table 9 indicates that, based on the intensity of pruritus, 40% of the cases exhibited mild symptoms, 35% exhibited moderate symptoms, and 25% exhibited severe symptoms.

**Table 9:** Distribution of patients according to the severity of pruritus in case groups

Severity of pruritus	No of Patients	Percentage
Mild	8	40
Moderate	7	35
Severe	5	25
Total	20	100

Table 10 exhibits the frequency distribution of patients in the control group according to their respective diagnoses, including androgenetic alopecia, callus, corn, hand and foot eczema, onychomycosis, oral lichen planus, palmoplantar keratoderma, prurigo nodularis, prurigo simplex, scabies, tinea corporis, tinea cruris, and tinea faciei. The data reveals that the aforementioned conditions were observed in 5% of the total study population. In contrast, eczema was detected in 10% of the study population, and vitiligo was the most prevalent condition, with a frequency of 20%.

**Table 10:** Distribution of patients according to diagnosis in the control group

Diagnosis	No of Patients	Percentage
Androgenetic alopecia	1	5
Callus	1	5
Eczema	2	10
Corn	1	5
Hand and foot eczema	1	5
Onychomycosis	1	5
Oral lichen planus	1	5
Palmoplantar keratoderma	1	5
Prurigo nodularis	1	5
Prurigo simplex	1	5
Scabies	1	5
Tinea corporis	1	5
Tinea cruris	1	5
Tinea facie	1	5
Vitiligo	4	20
Xerosis	1	5
Total	20	100

**Figure 1:**
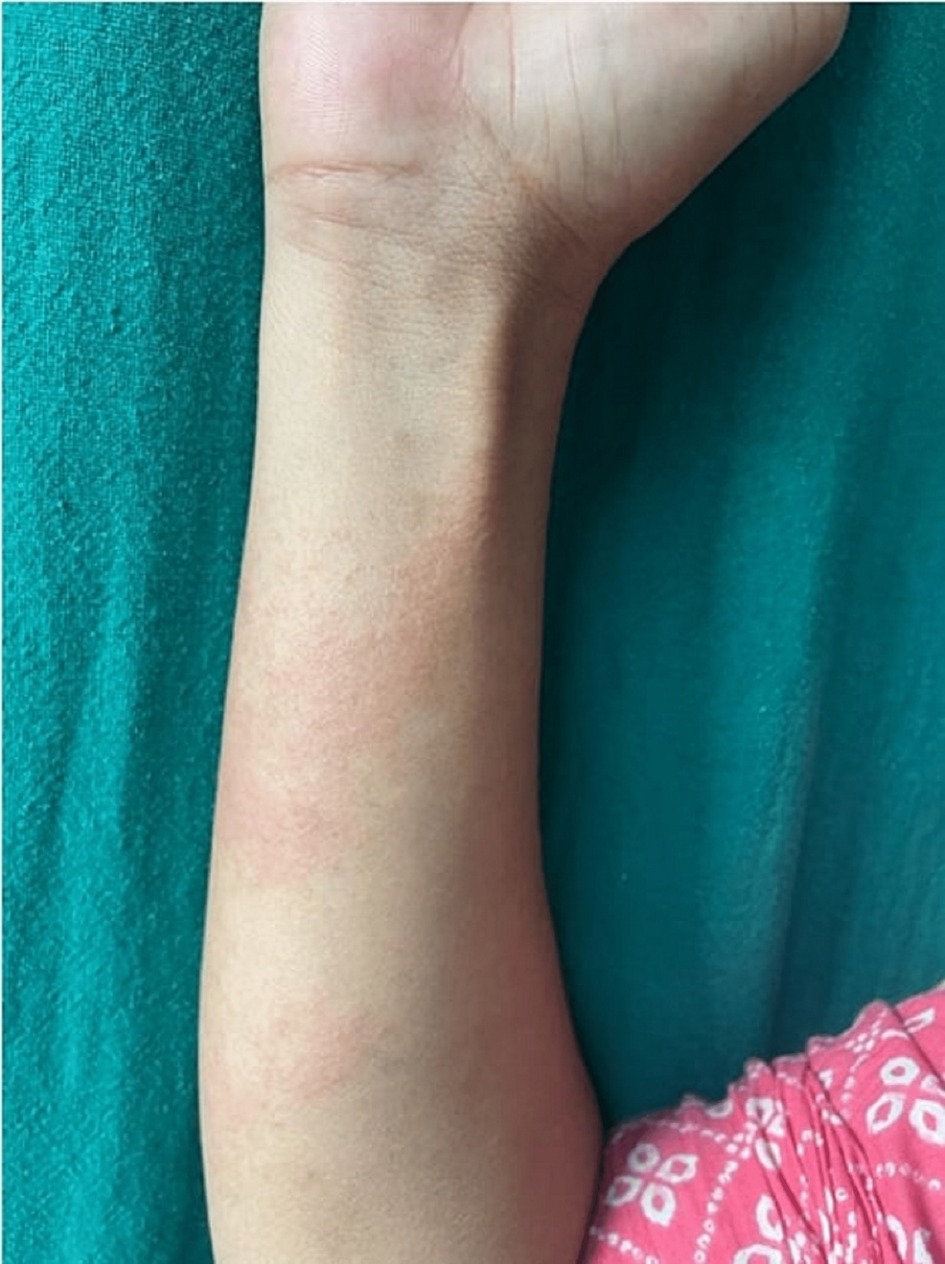
Patient presenting with less than 20 wheals in 24 hours with mild pruritus and dermatographism positive

**Figure 2:**
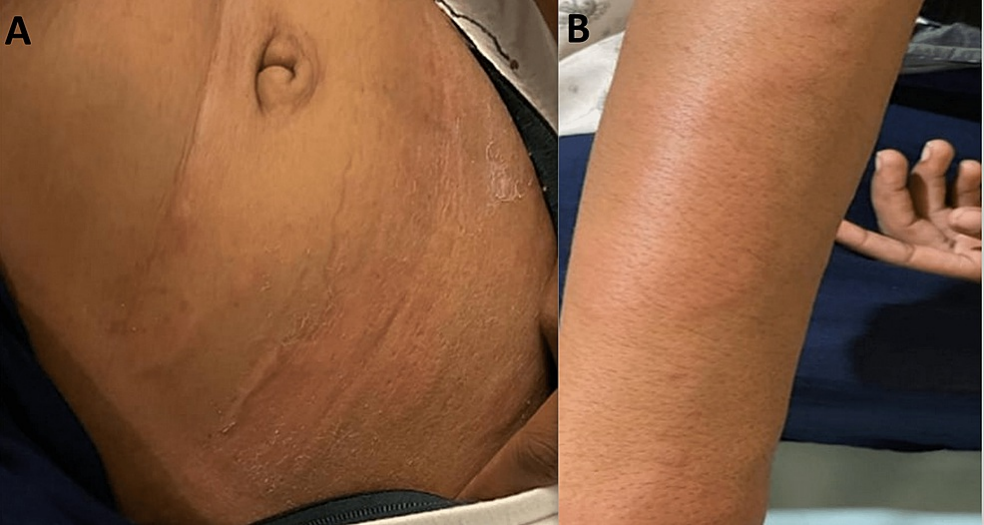
A, B) Patient presenting with 20-50 wheals in 24 hours with moderate pruritus and dermatographism positive

**Figure 3:**
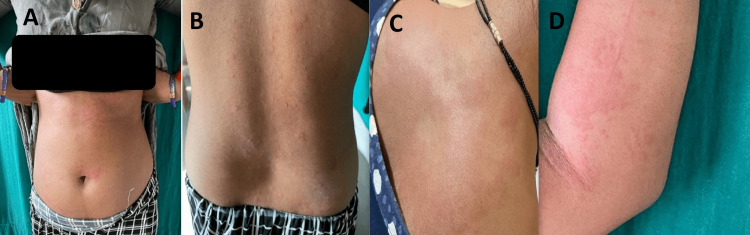
A, B, C, and D) Patient presenting with greater than 50 wheals in 24 hours with severe pruritus which is troublesome and interfering normal daily activities and sleep, dermatographism positive

Table 11 illustrates the distribution of individuals in the case group categorized by the number of wheals. The results indicate that 40% of the case group exhibited mild symptoms, less than 20 wheals. Additionally, 35% of the case group showed moderate symptoms, with 20 to 50 wheals. Finally, 25% of the case group displayed intense symptoms, with greater than 50 wheals. The mean±SD of the case group was calculated to be 28.45±23.19 (Figure 1-3).

**Table 11:** Distribution of patients according to the number of wheals in case groups

Number of wheels	No of Patients	Percentage
None	0	0
Mild (<20)	8	40
Moderate (20-50)	7	35
Intense (>50)	5	25
Total	20	100
Mean±SD	28.45±23.19(4-70 wheals/24 hrs.)	

## Discussion

The dataset analyzed in this study consists of measurements taken from 40 individuals, comprising 20 cases and 20 controls, between the ages of 20 and 60. The mean age for the case group was 36.40± 11.03 years, while for the control group, it was 32.95± 11.58 years.

Kasumagic-Halilovic et al. (2017) conducted a clinical trial involving patients and controls, with mean (SD) ages of 40.39 (±14.39) and 40.71 (±15.30) years, respectively, and a P-value of 0.896 [10]. The gender distribution in the case group revealed that 55% of the participants were female and 45% were male. In contrast, the control group had an equal percentage of male and female participants. In another study by Mariyath et al. (2017), out of 100 chronic urticaria patients, 75 were women and 25 were men, while the control group consisted of 12 men and 38 women [11].

Our present investigation estimated the triiodothyronine levels in both the case and control groups. Our results demonstrated that the triiodothyronine levels fell within the normal range for both groups. Similarly, Mariyath et al. (2017) [11] conducted a study on 100 patients with chronic spontaneous urticaria and 50 controls, wherein the levels of serum anti-TPO antibodies, triiodothyronine (T3), tetraiodothyronine (T4), and TSH were estimated. The results showed that 22% of the patients had abnormal T3 and T4 levels, whereas 22% and 12% of the controls had abnormal TSH levels. Furthermore, the estimated tetraiodothyronine values for the case and control groups in our current study resulted in a t-value of 0.80 and a p-value of 0.41, indicating that both groups had levels within the normal range. Nayaf et al. (2022) [12] conducted a review study comprising 50 patients with chronic spontaneous urticaria and 40 controls (without urticaria). The study did not reveal any significant difference in the mean serum tetraiodothyronine hormone levels between the patients and controls (P > 0.05).

Our investigation evaluated the case and control groups' thyroid-stimulating hormone (TSH) levels. The findings revealed that the TSH levels for both groups were within the normal range. The statistical analysis yielded a t-value of 2.19 and a p-value of 0.034 for the two groups. This finding is in contrast to a prior study by O'farrill-Romanillos et al. (2019) [13], which indicated that up to 54% of individuals with chronic spontaneous urticaria (CSU) exhibited a correlation with thyroid disease. Our study also found a significant result for anti-thyroperoxidase antibodies in both the cases and controls. Moreover, a study by Kasumagic-Halilovic et al. (2017) [10] revealed that 21 out of 70 individuals (30%) tested positive for anti-TPO.

In the case group, patients were evaluated for the severity of urticaria, with 40% demonstrating a score of one, 35% exhibiting a score of two, and 25% showing a score of three. The average duration of symptoms for patients with chronic spontaneous urticaria was calculated to be 6.90 ± 0.78 weeks, with 35% of cases lasting six weeks, approximately 40% lasting seven weeks, and 25% lasting eight weeks. Another study conducted by Swetha et al. in 2016 [14] revealed that only 46 (46%) out of 100 cases of chronic spontaneous urticaria investigated had a duration ranging from six weeks to five years [14].

Within the study group under investigation, 40% exhibited mild pruritus, 35% exhibited moderate pruritus, and 25% exhibited severe pruritus. Yosipovitch et al. (2002) [15] conducted a study with 100 patients, finding that 68 of them experienced daily pruritus, with the majority reporting severe pruritus that impeded their normal daily activities [15].

In our present study, patients in the control group were diagnosed with androgenetic alopecia, callus, corn, hand, and foot eczema, onychomycosis, oral lichen planus, palmoplantar keratoderma, prurigo nodularis, prurigo simplex, scabies, tinea corporis, tinea cruris, and tinea facie. Each contributed 5% of the study population, while eczema accounted for 10% and vitiligo for 20%.

According to our present study, patients in the case group were evaluated based on the number of wheals, with 40% displaying less than 20, indicating mild symptoms; 35% showing between 20 to 50 wheals, indicative of moderate symptoms; and 25% exhibiting over 50 wheals, suggesting intense symptoms. The mean±SD was calculated as 28.45±23.19. Alyasin et al. (2011) [16] reported a significant occurrence of wheals and large sizes in their study.

Limitations of the study

This is a hospital-based study and other autoimmune thyroid disorder markers are not taken, for example, anti-TSH receptor antibodies and thyroglobulin antibodies.

## Conclusions

Given that urticaria can significantly impact patients' quality of life, prompt disease management and patient involvement are essential. Due to the complexity of the condition, a tailored approach to management is necessary, involving trigger avoidance, treatment of related disorders, and pharmaceutical treatment of symptoms. Notably, there is a strong correlation between chronic spontaneous urticaria, thyroid autoimmune disease, and thyroid dysfunction. In particular, individuals with chronic spontaneous urticaria are more likely to test positive for TPO antibodies. Thus, from a clinical perspective, screening for thyroid autoimmunity should be considered in the presence of chronic spontaneous urticaria, regardless of the underlying cause. This study highlights the significant association between serum anti-TPO antibodies and chronic spontaneous urticaria. It underscores the importance of testing for these antibodies and the standard thyroid markers such as T3, T4, and TSH to minimize the long-term morbidity of this condition.
